# SARS-CoV-2-Specific T-Cell as a Potent Therapeutic Strategy against Immune Evasion of Emerging COVID-19 Variants

**DOI:** 10.3390/ijms251910512

**Published:** 2024-09-29

**Authors:** Keon-Il Im, Nayoun Kim, Junseok Lee, Ui-Hyeon Oh, Hye-Won Lee, Dong-Gun Lee, Gi-June Min, Raeseok Lee, Jinah Lee, Seungtaek Kim, Seok-Goo Cho

**Affiliations:** 1 Institute for Translational Research and Molecular Imaging, College of Medicine, The Catholic University of Korea, Seoul 06591, Republic of Korea; keonil1200@lucasbio.com (K.-I.I.); nkim@lucasbio.com (N.K.); poi03005@catholic.ac.kr (J.L.); beichest@catholic.ac.kr (G.-J.M.); 2 Research and Development Division, LucasBio Co., Ltd., Seoul 06591, Republic of Korea; ehoh@lucasbio.com (U.-H.O.); hwlee@lucasbio.com (H.-W.L.); 3 Division of Infectious Diseases, Department of Internal Medicine, Seoul St. Mary’s Hospital, College of Medicine, The Catholic University of Korea, Seoul 06591, Republic of Korea; symonlee@catholic.ac.kr (D.-G.L.); misozium03@catholic.ac.kr (R.L.); 4 Vaccine Bio Research Institute, College of Medicine, The Catholic University of Korea, Seoul 06591, Republic of Korea; 5 Department of Hematology, Seoul St. Mary’s Hematology Hospital, College of Medicine, The Catholic University of Korea, Seoul 06591, Republic of Korea; 6 Zoonotic Virus Laboratory, Institut Pasteur Korea, Seongnam 13488, Republic of Korea; jinah.lee@ip-korea.org (J.L.); seungtaek.kim@ip-korea.org (S.K.)

**Keywords:** coronavirus disease, immunotherapy, virus-specific T cells, viral immunity, severe acute respiratory syndrome coronavirus 2

## Abstract

Despite advances in vaccination and therapies for coronavirus disease, challenges remain due to reduced antibody longevity and the emergence of virulent variants like Omicron (BA.1) and its subvariants (BA.1.1, BA.2, BA.3, and BA.5). This study explored the potential of adoptive immunotherapy and harnessing the protective abilities using virus-specific T cells (VSTs). Severe acute respiratory syndrome coronavirus 2 (SARS-CoV-2) VSTs were generated by stimulating donor-derived peripheral blood mononuclear cells with spike, nucleocapsid, and membrane protein peptide mixtures. Phenotypic characterization, including T-cell receptor (TCR) vβ and pentamer analyses, was performed on the ex vivo-expanded cells. We infected human leukocyte antigen (HLA)-partially matched human Calu-3 cells with various authentic SARS-CoV-2 strains in a Biosafety Level 3 facility and co-cultured them with VSTs. VSTs exhibited a diverse TCR vβ repertoire, confirming their ability to target a broad range of SARS-CoV-2 antigens from both the ancestral and mutant strains, including Omicron BA.1 and BA.5. These ex vivo-expanded cells exhibited robust cytotoxicity and low alloreactivity against HLA-partially matched SARS-CoV-2-infected cells. Their cytotoxic effects were consistent across variants, targeting conserved spike and nucleocapsid epitopes. Our findings suggest that third-party partial HLA-matching VSTs could counter immune-escape mechanisms posed by emerging variants of concern.

## 1. Introduction

Coronavirus disease (COVID-19), caused by severe acute respiratory syndrome coronavirus 2 (SARS-CoV-2), presents symptoms like fever, chills, and dry cough, with severe cases leading to respiratory complications and death. High-risk groups, including older adults [1,2,3], those with blood cancers (e.g., malignant lymphoma) [4], and individuals on immunosuppressive regimens [5], are especially vulnerable. Impaired humoral immunity heightens the risk of severe disease and prolonged infection, leading to complications like pulmonary fibrosis and death, even after recovery from hematological disorders [6,7].

The continued genomic evolution of SARS-CoV-2 has exposed the limitations of vaccine-elicited humoral responses [8,9]. Variants like Omicron (BA.1/B.1.1.529), with augmented mutations in the spike protein receptor-binding domain, underscore the challenges of antibody-based strategies [10,11]. Although memory B cells adapt to new variants [12], the rapid emergence and significant mutations of variants of concern (VOCs) continue to challenge preventive measures and therapeutic strategies [13]. Although current therapeutic guidelines for COVID-19, encompassing immunomodulatory agents such as dexamethasone [14], antivirals (remdesivir [15], nirmatrelvir/ritonavir [16], molnupiravir) [17], and monoclonal antibodies [18], offer varying efficacy across disease stages, prolonged SARS-CoV-2 infections, notably in immunocompromised patients, remain challenging to treat.

The potential for immune escape and heightened disease virulence emphasizes the essential role of virus-specific T cells (VSTs) [19,20]. Recent studies have highlighted that the presence of memory T cells significantly influences the clinical course of, and recovery from, COVID-19 [2,21,22]. T cells play a pivotal role in viral elimination, particularly in patients with diminished humoral responses [23,24,25]. Memory T cells provide long-term protection even when antibody levels wane [26,27]. Correspondingly, the heightened presence of these cells is evident in individuals with milder clinical presentations than severe ones, where T-cell exhaustion and lymphopenia occur [28,29]. Prior research demonstrating the feasibility of expanding SARS-CoV-2 memory T cells from convalescent donors has been highlighted in various studies [30,31,32,33]. Additionally, a noteworthy breakthrough was demonstrated in adoptive cell therapy in a recent randomized (2:1), open-label, phase 1/2 trial. As reported by Papadopoulou et al. [34], this trial assessed the safety and efficacy of off-the-shelf, partially HLA-matched, convalescent donor-derived SARS-CoV-2-specific T cells (‘CoV-2-STs’). These observations highlight the protective capacity of VSTs, thereby setting the stage for therapeutic interventions that pivot on adoptive cell therapy.

To explore the potential of adoptive immunotherapy leveraging the protective abilities of VSTs, we established a minibanking system using VSTs from donors matched for at least one HLA class I allele. This strategy enables the rapid delivery of VSTs to COVID-19 patients, facilitating timely and effective treatment. We developed diverse VST products using various T-cell receptors (TCRs). Our findings indicate that these VSTs can effectively target and mediate cytotoxicity against both the ancestral SARS-CoV-2 and Omicron variants BA.1 and BA.5. Unlike vaccines which target the spike protein, VSTs recognize nucleocapsid and membrane proteins. This versatility, combined with the advantages of adoptive transfer and immediate availability from minibanks, highlights the potential of VST-based adoptive therapy for treating SARS-CoV-2.

## 2. Results

### 2.1. Enhancing SARS-CoV-2-Specific T-Cell Responses for Adoptive Immunotherapy: Phenotypic Characterization and TCR Diversity

**Figure 1:**
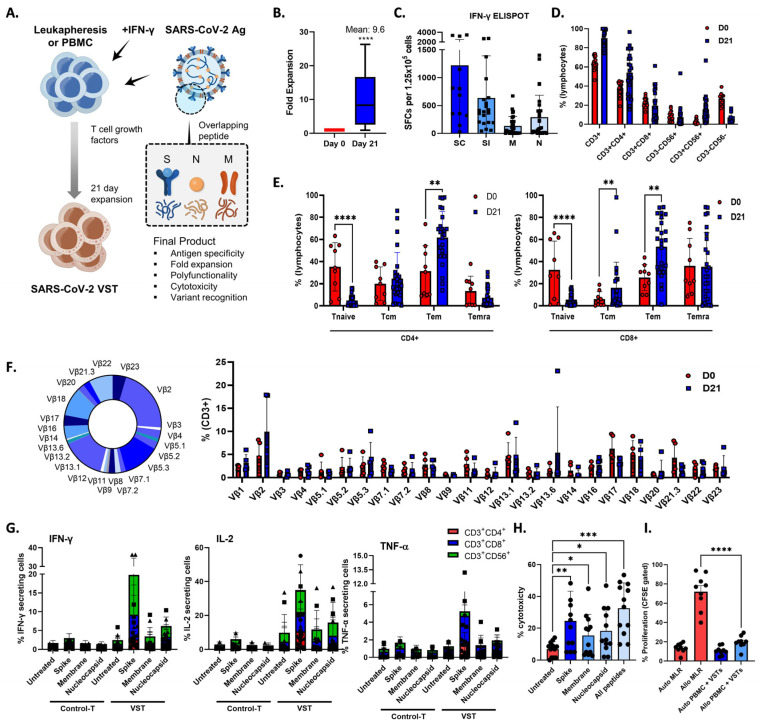
Enhancing SARS-CoV-2-specific T-cell (VST) responses for adoptive immunotherapy: phenotypic characterization and TCR diversity. (**A**) Representation of the 21-day VST expansion process, where PBMC or leukapheresis samples were stimulated with S, M, and N peptide pools. (**B**) Post-expansion fold increase in total cell count. (**C**) Post-expansion VST response against SARS-CoV-2 antigens. (**D**) Phenotypic distribution of expanded cell population, showing predominance of CD3^+^ T cells, with helper (CD3^+^CD4^+^), cytotoxic (CD3^+^CD8^+^), and NKT (CD3^+^CD56^+^) cell subsets. (**E**) Expression levels of T-cell memory markers, including central memory (CD45RA^−^/CD62L^+^), effector memory (CD45RA^−^/CD62L^−^), terminally differentiated effector memory (CD45RA^+^/CD62L^−^), and naïve markers (CD45RA^+^/CD62L^+^). (**F**) Multichannel flow-cytometric analysis of TCR vβ repertoire diversity in expanded cells, capturing >70% of all vβ chains and confirming the presence of all measurable vβ family members; representative donor (left) and summary data are shown as means ± SEMs (right). (**G**) Intracellular cytokine staining depicting antigen-specific IFNγ, IL-2, and TNF-α production in CD3^+^CD4^+^, CD3^+^CD8^+^, and CD3^+^CD56^+^ T-cell subsets in response to spike (S), nucleocapsid (N), and membrane (M) antigens. Minimal response in the absence of peptide stimulation underscores VST-activation specificity against SARS-CoV-2. Data are shown as SFC ± standard error of mean (SEM). (**H**) VST cytolytic activity toward carboxyfluorescein succinimidyl (CFSE)-labeled SARS-CoV-2 peptide-loaded autologous phytohemagglutinin-activated (PHA) blasts. Results show targeted lysis at an effector–target ratio of 50:1. (**I**) HLA-mismatched allogenic PBMC experiments confirm the absence of nonspecific autotargeting and alloreactivity that could lead to graft-versus-host disease; * *p* < 0.05; ** *p* < 0.01; *** *p* < 0.001; **** *p* < 0.0001. Scale and error bars indicate median and range, respectively. IFNγ: interferon gamma; M: membrane; N: nucleocapsid; PBMC: peripheral blood mononuclear cells; S: spike; SARS-CoV-2: severe acute respiratory syndrome coronavirus-2; SC: spike complete, SFC: spot-forming cell; SI, spike immunodominant; Tem: effector memory T cells; Tcm: central memory T cells; Temra: terminally differentiated effector memory T cells; TCR: T-cell receptor; VST: virus-specific T cell.

During a 21-day period, we exposed PBMCs or leukapheresis samples from donors to S, M, and N protein activators, thereby initiating the activation and expansion of VSTs that specifically target SARS-CoV-2 antigens (Figure 1A), which induced a 9.6-fold increase in the total cell count (Figure 1B), revealing a robust VST response-to-antigen stimulation (Figure 1C). We conducted a comprehensive examination of the phenotypic profile of the expanded cells. They were predominantly comprised of CD3^+^ T cells (90.1 ± 1.9%), including helper T cells (CD3^+^CD4^+^; 54.0 ± 4.7%), cytotoxic T cells (CD3^+^CD8^+^; 19.5 ± 3.5%), and NKT cells (CD3^+^CD56^+^; 7.4 ± 2.3%) (Figure 1D). Notably, high expression levels of central memory markers (CD45RA^−^/CD62L^+^; 20.4 ± 3.3%), effector memory markers (CD45RA^−^/CD62L^−^; 57.6 ± 3.4%), and terminally differentiated effector memory markers (CD45RA^+^/CD62L^−^; 21.3 ± 3.7%) were observed, whereas naïve markers (CD45RA^+^/CD62L^−^; 5.1 ± 0.7%) were expressed at lower levels (Figure 1E). Furthermore, to assess the diversity of the TCR vβ repertoire, we employed a multichannel flow cytometry panel capable of detecting >70% of all available vβ chains. All measurable vβ family members were present in the ex vivo-expanded cells (Figure 1F).

### 2.2. In Vitro Cytolytic Capacity of SARS-CoV-2-Specific T-Cell and Alloreactivity Assessment

To elucidate the T-cell subsets responsible for VST reactivity against S, N, and M antigens, we assessed IFNγ, IL-2, and TNF-α production within CD3^+^CD4^+^, CD3^+^CD8^+^, and CD3^+^CD56^+^ cell populations by gating followed by intracellular cytokine staining (Figure 1G). The antigen-specific release of IFNγ and IL-2 were predominantly detected in the CD3^+^CD8^+^ and CD3^+^CD56^+^ populations, whereas the CD3^+^CD4^+^ subset showed minimal response. Conversely, the TNF-α antigen-specific response was largely confined to the CD3^+^CD8^+^ subset. In contrast, control T cells cultured in the absence of peptide stimulation showed minimal antigen-specific cytokine production.

To investigate the in vitro cytolytic capacity of the VSTs, we co-cultured SARS-CoV-2-specific VSTs with CFSE-labeled SARS-CoV-2 peptide-loaded autologous PHA- blasts. The expanded VSTs specifically recognized and lysed SARS-CoV-2-peptide-loaded targets at an E:T ratio of 50:1 (Figure 1H). VST alloreactivity against both autologous and HLA-mismatched allogeneic PBMCs was absent in the CFSE proliferation assay (Figure 1I), suggesting a lower risk of inducing graft-versus-host clinical responses.

**Figure 2:**
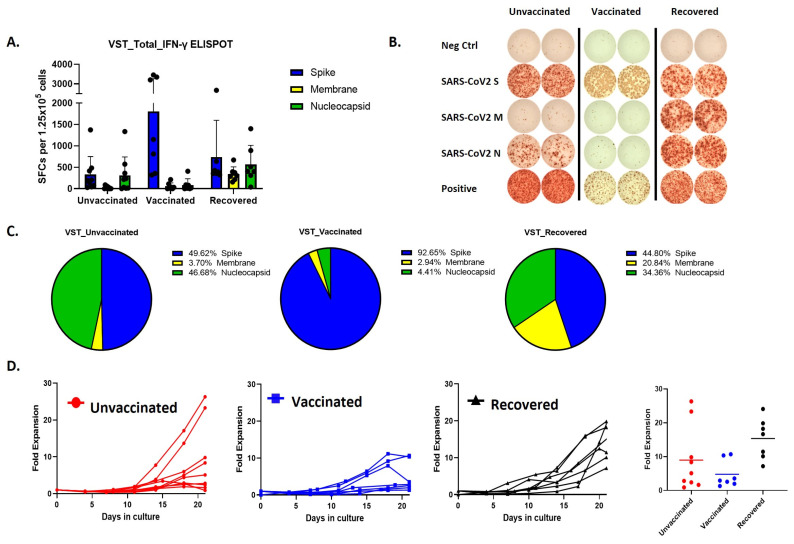
Comparative analysis of VST production and proliferation responses to viral peptides among groups based on SARS-CoV-2 exposure and vaccination status. (**A**,**B**) Evaluation of IFN-γ ELISPOT responses to spike, membrane, and nucleocapsid peptides using VSTs produced from donors in groups classified by SARS-CoV-2 exposure and vaccination status: unvaccinated (no prior infection), vaccinated (no prior infection), and recovered (post-infection). (**C**) IFN-γ response distribution in the unvaccinated group: Spike (49.62%), Nucleocapsid (46.68%), and Membrane (3.7%). IFN-γ response distribution in the vaccinated group: Spike (92.65%). IFN-γ response distribution in the recovered group: Spike (44.8%), Membrane (20.84%), and Nucleocapsid (34.36%). (**D**) Trends in proliferation rates and fold expansion after 10 and 17 days of incubation. IFN: interferon, SARS-CoV-2: severe acute respiratory syndrome coronavirus 2; VST: virus-specific T cell.

Remarkably, the predominant immune responses elicited by SARS-CoV-2 VSTs from individuals with differing vaccination statuses exhibited distinct patterns (Figure 2A,B). Specifically, vaccinated donors demonstrated a notable immune reaction, but the ELISPOT response was primarily directed against spike peptides whereas COVID-19-recovered individuals displayed a more balanced immune response targeting all three peptides (Figure 2C). Cells expanded from these donor groups also showed differences in the proliferation rates, where SARS-CoV-2 T cells expanded from COVID-19 recovered individuals showed the most fold expansion (Figure 2D).

### 2.3. Evaluating VST Cytotoxicity against SARS-CoV-2 Spike-Pseudotyped Lentivirus-Infected Cells

**Figure 3:**
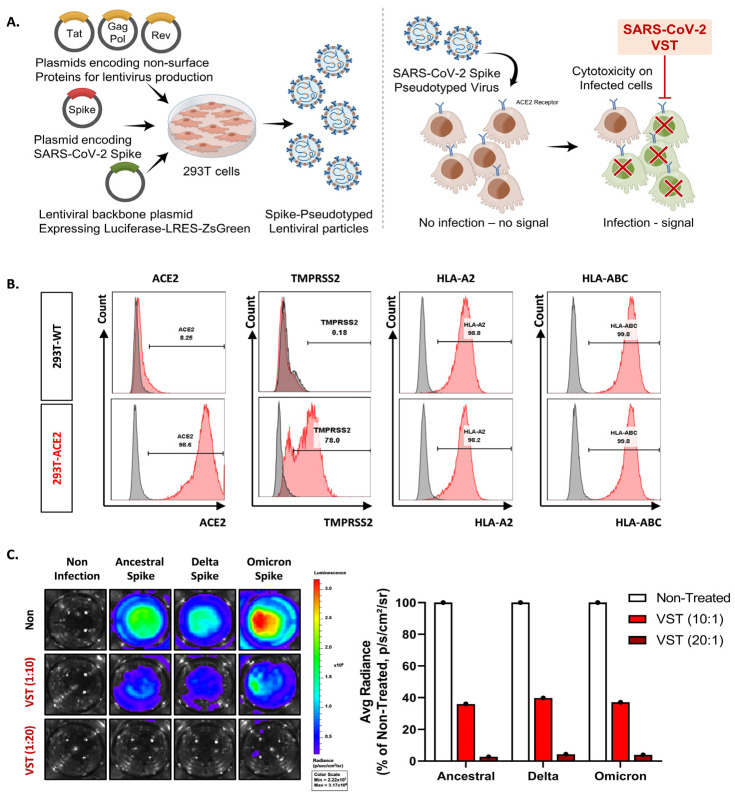
Evaluation of virus-specific T cell (VST) cytolytic capacity across SARS-CoV-2 spike variants. (**A**) An illustrative overview of the experimental setup and methodology including the generation and infection process of SARS-CoV-2 spike-pseudotyped lentivirus. (**B**) Flow cytometric characterization, highlighting pronounced expressions of ACE2, TMPRSS2, HLA *A02, and HLA-A/B/C in both HEK293 wild-type (293T-WT) and HEK293T-hACE2-TMPRSS2-mCherry (293T-ACE2) cells. (**C**) Bioluminescence analysis demonstrating a dose-dependent VST-induced cytotoxic response against cells infected by different variants of SARS-CoV-2 spike-pseudotyped lentiviruses. Induction of bioluminescence through luciferase is indicative of lentiviral infection, and a decrease in bioluminescence signals signifies significant VST-induced cytotoxicity toward the infected target cells. SARS-CoV-2: severe acute respiratory syndrome coronavirus 2.

**Figure 4:**
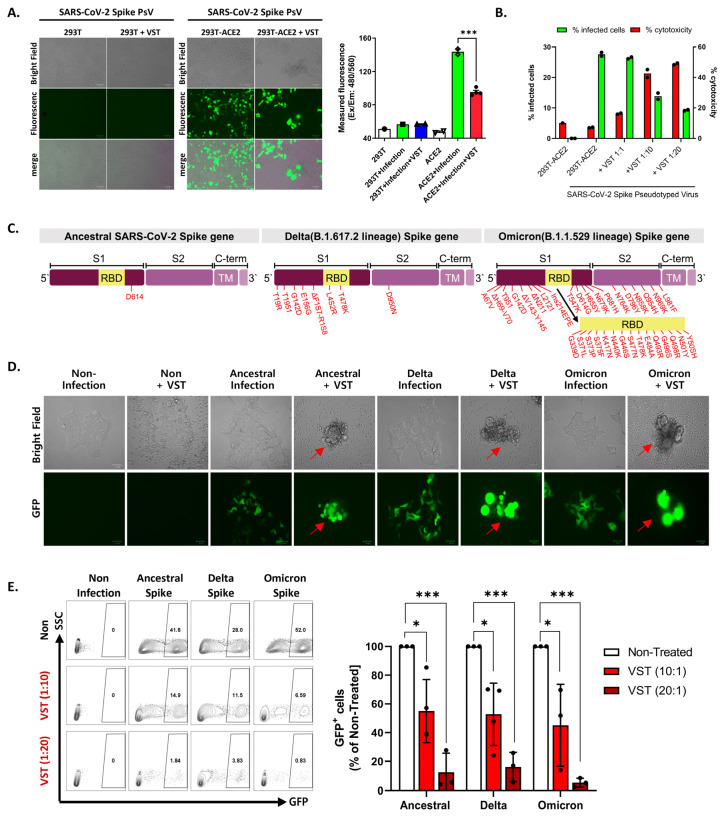
Evaluation of virus-specific T-cell (VST) reactivity and cytotoxicity across SARS-CoV-2 spike variants. (**A**) Fluorescence microscopy after 48 h co-culture of VST with HEK293T-hACE2-TMPRSS2-mCherry cells infected with SARS-CoV-2 spike-pseudotyped lentivirus. Green fluorescent protein (GFP) expression indicates lentiviral infection, whereas observations indicate significant VST-induced cytotoxicity toward infected, but not uninfected, target cells. (**B**) Flow cytometric analysis after VST co-culture shows a reduction in viable GFP-expressing cells, with an increase in the number of fixed-viability dye (FVD)-positive necrotic cells, which emphasizes the potent cytolytic capacity of VSTs. (**C**) Mutation sites within the spike protein for the Ancestral (D614), Delta (B.1.617.2), and Omicron (B.1.1.529/BA.1) variants are indicated by red arrows. (**D**) Fluorescence microscopic images of VST-induced cytotoxic effects against cells infected by different variants of SARS-CoV-2 spike-pseudotyped lentiviruses. The green color represents viral infection-induced GFP-expressing cells, whereas arrows indicate cells undergoing VST-induced cytolytic effects. (**E**) Flow cytometric analysis demonstrating dose-dependent VST-induced cytotoxic response against all evaluated spike variants. * *p* < 0.05; *** *p* < 0.001. HLA: human leukocyte antigen; IL: interleukin; INF: interferon; PBMC: peripheral blood mononuclear cell; SARS-CoV-2: severe acute respiratory syndrome coronavirus 2; SFC: spot-forming cell; TNF: tumor necrosis factor; RBD: receptor-binding domain; SARS-CoV-2: severe acute respiratory syndrome coronavirus 2; VST: virus-specific T cell.

We tested VSTs against HEK293T-hACE2-TMPRSS2-mCherry cells infected with SARS-CoV-2 spike-pseudotyped lentivirus. Target cells were transfected to overexpress human angiotensin-converting enzyme 2 (ACE2) (Figure 3A,B). We further confirmed the expression of transmembrane protease serine 2 (TMPRSS2) and HLA classes I and II. After 48 h co-culture, we conducted flow cytometry and bioluminescence analyses. The target cells exhibited GFP and bioluminescence expression upon infection with the pseudotyped lentivirus (Figure 3C). Fluorescence microscopy conducted 2 days post co-culture revealed that VSTs did not induce cytotoxicity in uninfected target cells, which remained viable. In contrast, VSTs demonstrated significant cytotoxicity against the infected target cells, leading to necrosis (Figure 4A,B). Flow cytometric analyses indicated a decline in the proportion of viable GFP-expressing target cells accompanied by an increase in the fraction of necrotic target cells that stained positive for FVD (Figure 4B). Furthermore, a dose-dependent cytotoxic effect was observed.

### 2.4. Cross-Recognition and Cytotoxicity of VSTs against SARS-CoV-2 Variants

To evaluate the potential targeting ability of these cells against clinically relevant viral variants, we examined their cross-reactivity to three distinct sequences of the SARS-CoV-2 spike protein: ancestral (D614), Delta variant (B.1.617.2), and Omicron variant (B.1.1.529/BA.1). Each vector carried mutations from the SARS-CoV-2 spike VOCs (Figure 4C). Given the intrinsic polyclonality and TCR diversity of our VSTs, we hypothesized that they would recognize each variant even in the absence of direct exposure to the corresponding antigens. When VSTs were co-cultured with cells infected with the SARS-CoV-2 spike-pseudotyped lentivirus representing these variant mutation sequences, they demonstrated notable cytotoxicity across all evaluated strains, confirmed by fluorescence microscopy (Figure 4D) and flow cytometry (Figure 4E).

### 2.5. Preserved Reactivity of the Polyclonal VST against SARS-CoV-2 Variants

**Figure 5:**
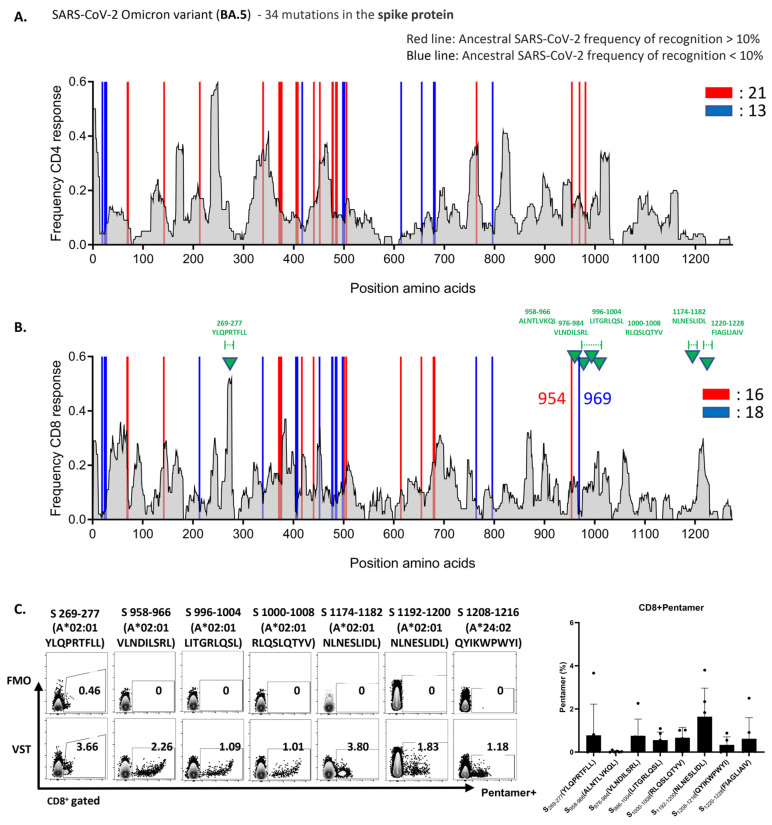
VSTs educated by the ancestral SARS-CoV-2 strain recognize conserved epitopes in the Omicron variant. The expected response frequency for the spike-mutation domain of Omicron BA.5, as inferred from the Immune Epitope Database, emphasizes the maintained high-response frequency even with the 34 present mutations. Analysis of spike-protein epitopes in the Omicron subvariant (BA.5) (**A**) targeted by CD4^+^ T cells (conservation rate: 82%), and (**B**) recognized by CD8^+^ T cells (conservation rate: 85%). (**C**) Representative data showcasing the pronounced reactivity of VSTs against seven specific Omicron BA.5 spike-protein immunogenic epitopes (indicated by green triangles in (**B**)), determined using pentamer analysis. A bar graph consolidates the results from 10 donors. FMO: fluorescence minus one; SARS-CoV-2: severe acute respiratory syndrome coronavirus 2; VST: virus-specific T cell.

To uncover the underlying mechanism of the robust VST response to the Omicron variant BA.5, we analyzed variant-specific epitopes. Spike-protein epitopes targeted by CD4^+^ and CD8^+^ T cells were conserved at rates of 82% and 85% (Figure 5A,B) and those of nucleocapsid epitopes at 90% and 93%, respectively (Supplementary Figure S1A). For membrane epitopes, both cell types showed a 96% conservation rate (Supplementary Figure S1B). Furthermore, we assessed the reactivity of the VST expanded using a peptide mixture derived from the reference strain (NC_045512.2) against Omicron variant peptides. These mutations did not compromise the VST antigen-specific responses. Figure 5C illustrates the pronounced reactivity of the VST to seven distinct immunogenic epitopes of the spike antigen via pentamer analysis. Remarkably, all 10 donors exhibited consistent reactivity against distinct immunogenic epitopes and diverse mutant spike peptides, highlighting the potential of the polyclonal VST to efficiently target mutations, thereby reducing the possibility of immune escape following T-cell therapy.

### 2.6. VST Activation and Targeted Cytotoxicity Toward Cells Expressing SARS-CoV-2 Spike and Nucleocapsid Proteins

**Figure 6:**
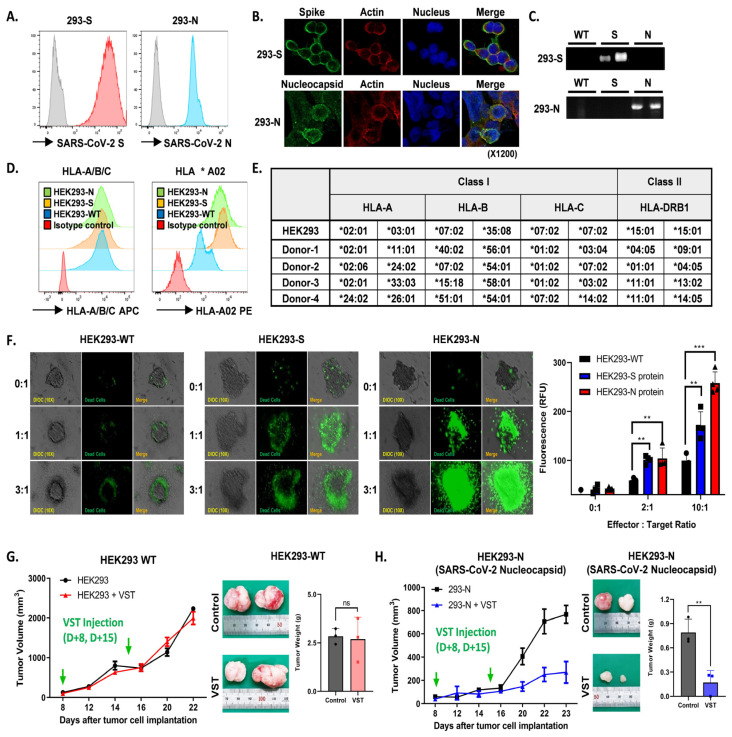
Antigen-specific activation and targeted cytotoxicity toward cells expressing SARS-CoV-2 spike and nucleocapsid proteins. (**A**) Flow cytometric analyses of SARS-CoV-2 spike (S) and nucleocapsid (N) protein expression in engineered cell lines. (**B**) Confocal imaging visually confirms S and N protein expressions. (**C**) Western blotting validates S and N protein expressions. (**D**) Flow cytometric characterization showing pronounced HLA-A/B/C and HLA *A02 expressions across HEK293 wild-type (WT), HEK293 S, and HEK293 N cells. (**E**) Effector VSTs from a donor with matching HLA class I allele relative to target 293T cells. (**F**) Cell Tox Green assay demonstrating increased cytotoxic susceptibility in S protein-expressing HEK293 cells, with cytotoxicity against N protein-transduced HEK293 cells. (**G**) Post-administration tumor-growth pattern in a xenograft mouse model inoculated with HEK293-WT cells. Green arrows indicate the time points of VST injection. VSTs neither recognize nor exert cytotoxic effects on SARS-CoV-2 antigen-unmodified WT cells, leading to continued tumor growth. (**H**) With HEK293-N cells expressing the SARS-CoV-2 N protein in the xenograft model, green arrows indicate the time points of VST injection. VSTs exhibit specific recognition and potent cytotoxicity, resulting in significant suppression of tumor growth. ** *p* < 0.01; *** *p* < 0.001. HLA: human leukocyte antigen; SARS-CoV-2: severe acute respiratory syndrome coronavirus 2; VST: virus-specific T cell.

HEK293 cell lines modified to express either SARS-CoV-2 S or N proteins consistently showed robust protein expression, confirmed by flow cytometry (Figure 6A), confocal imaging (Figure 6B), and Western blotting (Figure 6C). Flow cytometric analysis also revealed significant HLA-A/B/C and HLA *A02 expression across all cell lines (Figure 6D). Effector VSTs from donors with at least one HLA class I allele matching the target 293T cells demonstrated antigen-specific responsiveness (Figure 6E), displaying spatial heterogeneity in co-culture, including active peripheral proliferation, transient medial quiescence, and central hypoxia. Enhanced cytotoxicity was observed in SARS-CoV-2 spike-expressing HEK293 cells compared to WT cells in the Cell Tox Green assay, with similar results seen in N protein-transduced HEK293 cells (Figure 6F). In a parallel assay, VSTs stained with PKH26 Red were co-incubated with target cells, showing increased fluorescence in SARS-CoV-2 protein-transduced HEK293 cells after 24 h, indicative of VST antigen-triggered expansion. Live cell imaging demonstrated efficient elimination of HEK293 N cells by antigen-specific VSTs, evidenced by reduced CFSE-labeled target cells and increased activity of PKH26 Red-labeled T cells. In contrast, naïve T cells exhibited no cytotoxicity against HEK293 N cells, remaining in the periphery of the spheroid and eventually dissipating (Supplementary Figure S2). Finally, in a tumor xenograft model, inoculation with HEK293-WT or HEK293 N cells at 1-week intervals showed continued tumor growth for HEK293-WT cells (Figure 6G), while HEK293 N cells displayed a substantial reduction in tumor volume and weight (Figure 6H).

### 2.7. Cytotoxic Activity of VSTs on Cells Infected with Authentic SARS-CoV-2 Omicron Mutants

**Figure 7:**
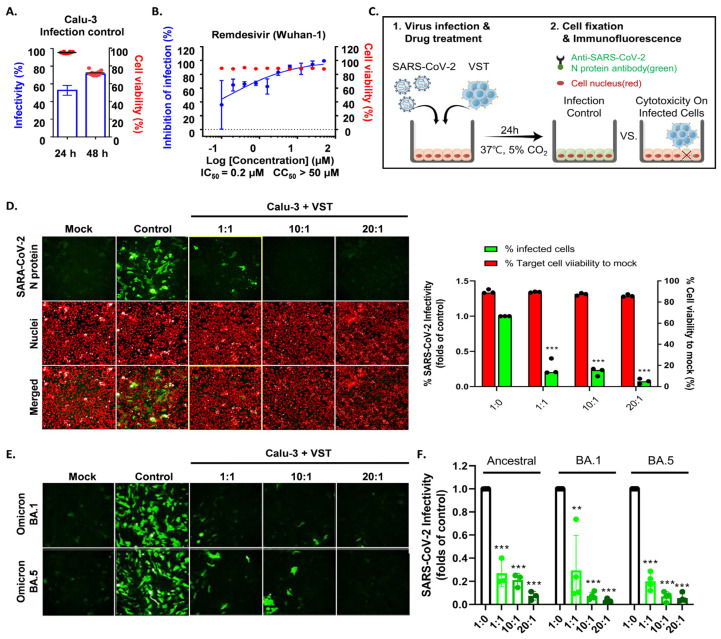
Cytotoxic activity of VST against SARS-CoV-2 Omicron mutant-infected Calu-3 cells. (**A**) Comparison of SARS-CoV-2 infectivity in Calu-3 cells at 24 and 48 h post-infection. Data indicate increased infectivity and significant cytopathic effects at the 48 h timepoint. (**B**) Antiviral activity of remdesivir against SARS-CoV-2 in Calu-3 cells, serving as a positive control for the suppression of viral replication. (**C**) Visualization of SARS-CoV-2 N protein in Calu-3 cells after a 24 h co-culture period with VSTs, indicating viral infection. (**D**) Dose-dependent cytotoxic activity of VSTs against SARS-CoV-2-infected Calu-3 cells, showcasing the ability of VSTs to selectively target and eliminate virus-infected cells. (**E**) Efficacy of VSTs derived from four different donors against the Omicron mutant strains, NCCP43408 and NCCP43426, revealing a consistent and potent dose-dependent cytotoxic response. (**F**) Reflective comparison of the potency of VSTs against the ancestral SARS-CoV-2 strain, emphasizing the consistent therapeutic potential across virus variants. ** *p* < 0.01; *** *p* < 0.001. SARS-CoV-2: severe acute respiratory syndrome coronavirus 2; VST: virus-specific T cell.

**Figure 8:**
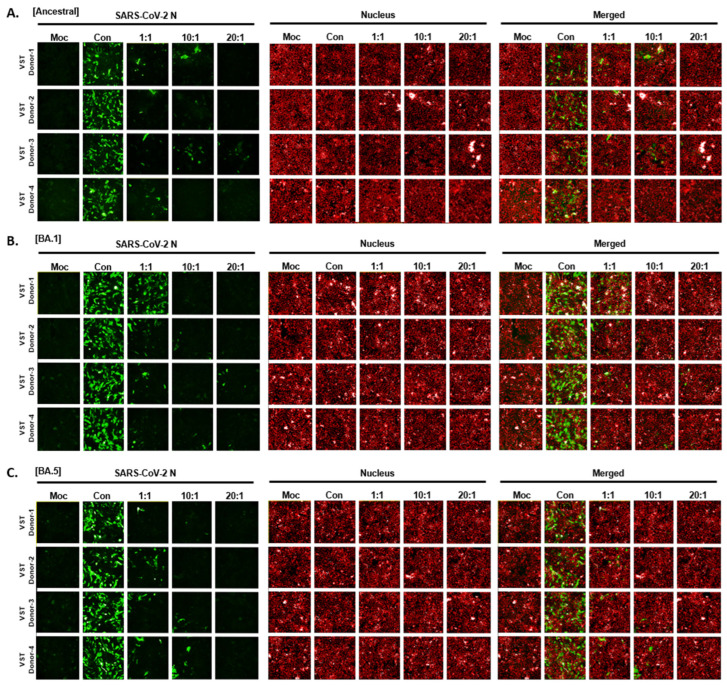
Cytotoxic activity of VST on cells infected with authentic SARS-CoV-2 Omicron mutants. (**A**) Dose-dependent response against the ancestral SARS-CoV-2 strain. (**B**) Dose-dependent response against the SARS-CoV-2 BA.1 variant. (**C**) Dose-dependent response against the SARS-CoV-2 BA.5 variant. N, nucleocapsid; SARS-CoV-2: severe acute respiratory syndrome coronavirus 2; VST: virus-specific T cell.

To determine if VSTs could target and eliminate cells infected with authentic SARS-CoV-2, human lung epithelial cells (Calu-3) were infected and co-cultured at 24 and 48 h. Immunofluorescence staining of the SARS-CoV-2 N protein identified infected cells, while nuclear staining quantified the total cell population. At 24 h, infection rates in Calu-3 cells ranged from 48 to 60%, with cell viability over 97% and no cytopathic effects. By 48 h, infection increased to 70–80% with notable cytopathic effects and lower cell survival (Figure 7A). Hence, we selected 24 h for co-culture of VST effector cells with SARS-CoV-2-infected Calu-3 cells. Remdesivir, a known SARS-CoV-2 replication inhibitor, was used as a control drug and demonstrated antiviral activity against SARS-CoV-2 in Calu-3 cells (Figure 7B). After a 24 h co-culture, we washed away the VSTs and exclusively analyzed the adherent virus-infected Calu-3 cells by immunostaining cell nuclei and SARS-CoV-2 N protein (Figure 7C). Remarkably, VSTs exhibited a dose-dependent cytotoxic effect and effectively recognized and eliminated virus-infected cells (Figure 7D). VSTs from four donors also showed strong, dose-dependent cytotoxicity against Omicron variants NCCP43408 and NCCP43426 (Figure 7E), similar to their effect on ancestral SARS-CoV-2 (Figure 7F and Figure 8).

## 3. Discussion

We generated SARS-CoV-2-specific T cells (VSTs) using peptide pools of 15-mers with an overlap of 11 amino acids derived from the ancestral SARS-CoV-2 strain. Initially, VSTs were produced using the ancestral SARS-CoV-2 sequence peptides before the emergence of the known VOCs major variants. These VSTs showed strong cross-reactive cytotoxic responses against conserved antigen epitopes across several SARS-CoV-2 variants, including Alpha, Beta, Gamma, Delta, and Omicron (Figure 5).

Therapies targeting the SARS-CoV-2 spike protein can lose potency due to mutations like N501Y and E484K (Table 1), which increase transmissibility and resistance [12,35]. When we analyzed the SARS-CoV-2 proteome, specifically focusing on the spike glycoprotein, our findings showed that approximately 82% of MHC class II (CD4^+^ T cell) epitopes and 85% of MHC class I (CD8^+^ T cell) epitopes of these proteins in the VSTs were conserved. Notably, a significant proportion of T-cell epitopes in the IEDB corresponding to these structural proteins were preserved across all examined variants (Figure 5A,B). The findings of recent studies align with our findings on ex vivo-expanded VSTs [36,37] that, unlike variable neutralizing antibody epitopes, T-cell epitopes targeted by VSTs, both endogenous and ex vivo, are abundant. These conserved regions serve as primary targets for SARS-CoV-2-specific T-cell responses, emphasizing the potential of VST-based therapeutic approaches against novel and forthcoming mutations.

In addition, we generated VSTs using the spike immunodominant (SI) peptide pool, which was derived from the predicted immunodominant sequence of SARS-CoV-2, and the 15-mer overlapping spike complete (SC) peptide pool, with an overlap of 11 amino acids, spanning the entire length of the spike protein. We then conducted ELISPOT analysis between SI-VSTs and SC-VSTs, revealing that SC-VSTs were more abundant and had a stronger response to mutant peptides, indicating superior recognition of multiple epitopes (Supplementary Figure S3). This strategy of producing polyclonal VSTs covering the full viral sequence is promising given the risk of new mutations.

While the spike protein mutates under pressure, internal proteins like the N protein remain more conserved (Supplementary Figures S4 and S5) [38]. The Omicron BA.5 subvariant had mutations at 65 sites, with the spike protein affected most (ORF, 20; spike, 34; membrane, 3; envelope, 1; and nucleocapsid, 7). In particular, the spike protein was most frequently altered, with mutations spanning 34 areas, whereas mutations in the nucleocapsid were limited to seven regions (Supplementary Figure S3A). Further analysis of the SARS-CoV-2 nucleocapsid protein proteome revealed that 90% of MHC class II and 93% of MHC class I epitopes in the nucleocapsid protein were conserved. In the context of the expected binding affinity frequency for the BA.5 nucleocapsid mutation, a notable frequency of >10% was observed in one of the seven mutated regions in the CD4^+^ T-cell epitope. For the CD8^+^ T-cell epitope, four of the seven regions predicted a frequency below 10%, and the recognition of the other three regions remains unknown.

Most of these epitopes, despite some mutations, remain targets for VSTs. Our pentamer assay confirmed VST responses in the N 223-231 (A02:01 LLLDRLNQL) and N 362-370 (A11:01 KTFPPTEPK) regions (Supplementary Figure S6). Therapies targeting N proteins like VSTs may offer broader, more stable protection as the virus becomes endemic.

Our study confirmed VST efficacy in vitro against cells infected with authentic SARS-CoV-2. In a BSL-3 setting, VSTs recognized and eliminated infected cells in a dose-dependent manner, showing consistent effectiveness across Omicron variants and ancestral strains (Figure 7). A key strength is our dual approach, using both SARS-CoV-2 spike-pseudotyped viruses (Figure 4) and authentic viruses (Figure 7). While pseudotyped viruses help test antibodies or drugs, they may not fully reflect VST recognition of multiple viral components beyond the spike protein, such as nucleocapsid and membrane proteins. This approach more holistically addresses VST cytotoxicity, underscoring the significance of our findings for future SARS-CoV-2 therapies.

Animal models of COVID-19 have aided in studying pathogenesis and treatments [39,40,41] but do not fully replicate human disease. Evaluating human T cells in animals is complicated by the MHC system. Sefik et al. [42] developed a humanized mouse model by engrafting human stem cells and inducing ACE2 expression before infecting mice with authentic SARS-CoV-2, mimicking COVID-19 immunopathogenesis. In contrast, we used SARS-CoV-2 protein-expressing HEK293 cells in immunodeficient mice to show T-cell recognition of target antigens via MHC and TCRs. Although limited in assessing full immune system interactions, this model demonstrated our T-cell products’ ability to recognize and kill SARS-CoV-2 antigen-expressing cells in vivo (Figure 6G,H). While more humanized models are needed, VSTs have been safely used in clinical trials for viral diseases, supporting their use in early-phase trials for emerging viruses.

**Figure 9:**
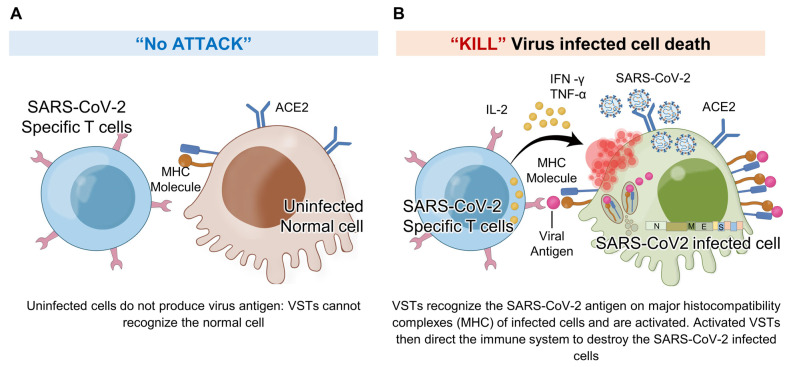
VSTs against SARS-CoV-2 infected cells: In response to SARS-CoV-2 infection, virus-specific T cells (VSTs) specifically target cells including epithelial cells and macrophages that become infected and express the ACE2 receptor. These cells, once infected, process and present viral antigens, which VSTs recognize through TCR-MHC I interactions. This detection triggers VSTs to release cytotoxic granules containing interferon-gamma (IFN-γ) and granzyme B, leading to the effective elimination of the infected cells and impeding the virus’s replication process. Notably, VSTs demonstrate specificity in their response: (**A**) they do not show cytotoxicity toward normal, uninfected cells, as these do not present SARS-CoV-2 antigens on their surface, and (**B**) they actively exhibit cytotoxicity against cells expressing virus antigens, thereby effectively inhibiting the viral replication cycle. IL: interleukin; MHC: major histocompatibility complex; SARS-CoV-2: severe acute respiratory syndrome coronavirus 2; TCR: T cell receptor; TNF: tumor necrosis factor.

Our study successfully generated SARS-CoV-2 VSTs capable of targeting a wide range of antigens, including ancestral strains and variants like Omicron BA.1 and BA.5 (Figure 9). The analysis confirmed a diverse TCR vβ repertoire and consistent cytotoxicity across strains, underscoring the VSTs’ ability to target conserved viral epitopes. Given the increased risk for high-risk groups, including older adults, blood cancer patients, and those on immunosuppressive therapies, we launched a clinical study to assess the safety and efficacy of these VSTs in vulnerable COVID-19 patients (CRIS Registration No. KCT0008222). This suggests that third-party, partially HLA-matched VSTs could help counter immune evasion in emerging variants and benefit the most vulnerable populations.

## 4. Materials and Methods

### 4.1. Study Design

#### 4.1.1. Donors

Donors were recruited to provide either 50 mL of whole blood or one blood volume via leukapheresis. The cohort included 11 recovered COVID-19 patients, four with ongoing moderate COVID-19, and 25 randomly selected unexposed donors. Informed consent was obtained (Seoul St. Mary’s Hospital IRB approval nos. KC20TSSI0274, KC20TSSI0872; Clinical Research Information Service, Republic of Korea (nos. KCT0005370, KCT0005864)), following the Declaration of Helsinki. Recovered donors had no fever, showed clinical improvement without antipyretics, and tested negative for SARS-CoV-2 on two PCR tests at least 24 h apart. Unexposed donors reported no symptoms and were IgG-negative for SARS-CoV-2. HLA typing was performed at the Catholic Hematopoietic Stem Cell Bank by sequence-based methods as previously described [43]. Donor characteristics are detailed in Table 2 and Supplementary Table S1.

#### 4.1.2. SARS-CoV-2-Specific T-Cell (VST) Generation

Peripheral blood mononuclear cells (PBMCs) were isolated via Ficoll gradient centrifugation. A minimum of 1 × 10^6^ PBMCs were seeded at a density of 1 × 10^7^ cells/mL and stimulated with peptivators for SARS-CoV-2 spike (S; Cat. No. 130-126-701), membrane (M; Cat. No. 130-126-703), and nucleocapsid (N; Cat. No. 130-126-699) proteins (1 μg/mL; Miltenyi Biotec, Bergisch Gladbach, Germany). A peptivator is a pool of lyophilized peptides comprising 15-mer sequences with 11 overlapping amino acids, covering the entire sequence of each protein. The S protein peptivator covers only the immunodominant sequence domains of the spike glycoprotein. On the same day, the cells were stimulated with 50 ng/mL of recombinant human interferon-gamma (rhIFN-γ; R&D Systems, Minneapolis, MN, USA, Minneapolis, MN, USA). A few days later, the cells were expanded using recombinant human interleukin-2 (60 ng/mL; R&D Systems) every 3–4 days for 3–4 weeks. The cells were maintained in AIM-V medium (Gibco, Thermo Fisher Scientific, Wilmington, DE, USA) with 5% human serum (Sigma-Aldrich, St. Louis, MO, USA) at 37 °C and 5% CO_2_, harvested, and cryopreserved.

#### 4.1.3. Immunophenotyping

Immunophenotyping was conducted using fluorescence-conjugated antibodies against CD3 (UCHT1), CD4 (SK3), CD8 (SK1), CD14 (61D3), CD16 (CB16), CD19 (HIB19), CD45RA (H100), CD45RO (UCHL1), CD56 (TULY56, CMSSB), and CD57 (QA17A04; Biolegend, San Diego, CA, USA) and CD62L (DREG-56). All antibodies were purchased from eBioscience, Inc. (San Diego, CA, USA), unless mentioned otherwise. Cells were stained, washed, and analyzed via flow cytometry, with gating strategies based on low forward scatter (FSC) and low side scatter (SSC) gating. The T-cell markers CD3, CD4, and CD8 and the NK cell marker CD56 were used to identify the major lymphocyte subsets. Various markers have been used to assess cell activation, cytokine production, and cell exhaustion.

#### 4.1.4. Intracellular Cytokine Staining after Peptide Stimulation

Expanded cells were stimulated overnight with SARS-CoV-2 S, M, and N peptivators (1 μg/mL each; Miltenyi Biotec). Protein transport inhibitors containing monensin (BD GolgiStop; Pharmingen, San Diego, CA, USA) inhibitors were added during the last 4 h of incubation at 37 °C, under 5% (*v*/*v*) CO_2_. Positive controls were stimulated with phorbol 12-myristate 13-acetate (PMA), ionomycin, brefeldin A, and monensin (Invitrogen Corp., Waltham, MA, USA); negative controls were left unstimulated. Surface markers were stained for CD3, CD56, CD8, and CD4, followed by intracellular IFNγ, TNF-α, and IL-2 staining according to the manufacturer’s instructions (eBioscience). Cells were analyzed using a Fortessa Flow Cytometer (BD Biosciences, San Jose, CA, USA).

### 4.2. Cytotoxicity Assay of VSTs by Peptide-Pulsed Phytohemagglutinin (PHA)-Blasts

#### 4.2.1. Preparation of PHA-Blasts

PHA-blasts were generated by stimulating autologous PBMCs with PHA (3 μg/mL; Sigma-Aldrich) and rhIL-2 (25 IU/mL) in AIM-V medium with 5% human serum for 3–4 days. For peptide pulsing, PHA-blasts (1 × 10^7^/mL) were incubated with SARS-CoV-2 S, M, and N protein peptivators (1 μg/mL; Miltenyi Biotec) for 2 h at 37 °C and 5% (*v*/*v*) CO_2_.

#### 4.2.2. Cytotoxicity Assay

Flow cytometric cytotoxicity assays were conducted as previously described [44]. Target cells (1 × 10^6^/mL) were labeled with carboxyfluorescein succinimidyl ester (CFSE) in a complete culture medium at room temperature under 5% CO_2_ for 20 min according to the manufacturer’s protocol. Target cells (1 × 10^5^) were incubated with effector cells at various effector–target ratios overnight. Prior to flow cytometry, cells were stained with 7-aminoactinomycin D (7-AAD; eBioscience), and cytotoxicity was measured using a FACS Canto (BD Biosciences). Target cells were gated on CFSE+ cells and examined for cell death based on 7-AAD uptake. The percentage of effector cell-mediated cytotoxicity was calculated using the following equation:

$$Cytotoxicity\;(\%)=\frac{\left(Dead\;target\;cells\left(\%\right)-spontaneous\;deaths\left(\%\right)\right)\times 100}{\left(100-spontaneous\;deaths\left(\%\right)\right)}$$

### 4.3. Generation of SARS-CoV-2-Spike-Pseudotyped Lentivirus

The spike protein sequence (Spike Ancestral, Delta, Omicron) of the SARS-CoV-2-pseudotyped vector (Spike Ancestral, Cat. No. plv-spike; Delta, Cat. No. plv-spike-v8; Omicron, Cat. No. plv-spike-v11; InvivoGen, San Diego, CA, USA) was codon-optimized for human expression. A replication-deficient lentivirus backbone expressing GFP and luciferase was used. This configuration allows bioluminescence induction through oxidation of luciferin, a substrate for luciferase. 293T cells were transfected with the pseudotyped vector and backbone using BioT transfection reagent (Cat. No. B01-01, Bioland Scientific, Paramount, CA, USA). After 72 h, the supernatant was centrifuged at 500× *g* for 5 min and then passed through a 0.45 μM, filtered, and concentrated with a lentivirus concentrator (Cat. No. TR30026, Origene, Rockville, MD, USA). The resultant virus pellet was resuspended in phosphate-buffered saline and subsequently stored at −80 °C. The titre of the prepared SARS-CoV-2-spike-pseudotyped lentivirus was ascertained via the Lenti-X™ qRT-PCR Titration kit (Cat. No. 631235, Takara, Shiga, Japan). All procedures were performed in a BSL-2 laboratory.

### 4.4. Infectivity and Cytotoxicity Analyses of SARS-CoV-2-Spike-Pseudotyped Lentivirus

HEK293T-hACE2-TMPRSS2-mCherry cells (target cells; Cat. No. NR-55293, BEI Resources, Manassas, VA, USA) were seeded at 3 × 10^3^ cells per well in 96-well plates and incubated for 24 h. Subsequently, the SARS-CoV-2 spike-pseudotyped lentivirus was introduced according to the previously determined S protein titres. For cytotoxicity evaluation, VST cells were used as effector cells with effector–target (E:T) ratios of 0:1, 1:1, 10:1, and 20:1. After the addition of VSTs, the co-culture was maintained for 2 days, after which the cells were collected, rinsed with staining buffer, and subsequently labeled with fixed viability dye (FVD) eFluor™ 780 (Invitrogen, Waltham, MA, USA) for 30 min at 4 °C. After staining, the cells were washed and resuspended in the staining buffer. Flow cytometric analysis was performed on a FACS_LSR Fortessa (BD Pharmingen, San Diego, CA, USA), and data were processed using FlowJo v10.8.1 (TreeStar). Cytotoxicity results are presented as the percentage of FVD-positive necrotic target cells or viable GFP-expressing target cells.

### 4.5. Cytotoxic Effect of VSTs on Authentic SARS-CoV-2

To assess the cytotoxic response of VSTs to live SARS-CoV-2, Calu-3 cells were plated at 1.0 × 10^5^ cells per well in 96-well μClear plates (Greiner Bio-One, Kremsmünster, Austria) with Eagle’s Minimum Essential Medium containing 20% fetal bovine serum (FBS), 1× MEM Non-Essential Amino Acids, and 1× antibiotic-antimycotic solution (Gibco, Thermo Fisher Scientific, Waltham, MA, USA), 24 h before infection with ancestral SARS-CoV-2 (NCCP43326) and Omicron subvariants BA.1 (NCCP43408) and BA.5 (NCCP43426). The multiplicities of infection were calibrated for cell viability >90% and virus infectivity >65% at 0.6, 1.0, and 2.6, respectively, for ancestral SARS-CoV-2, Omicron BA.1, and Omicron BA.5. At 24 h post-infection, the cells were fixed with 4% paraformaldehyde and permeabilized with 0.25% Triton-X100. Immunofluorescence staining of the N protein was performed to quantify cell counts and infection rates using Operetta CLS (PerkinElmer) with Harmony software, version 4.8 [45]. Statistical analysis of VST-induced cytotoxicity was performed using Student’s *t*-test. Remdesivir was used as a control for antiviral activity, and the results were standardized against mock-infected (0% infection) and negative control (100% infection, treated with 0.5% dimethyl sulfoxide) cells on each assay plate. Data were normalized and analyzed using dose–response curve fitting and confirmed in duplicate, which was supported by the computation of Z’-factor and coefficient of variation. All authentic SARS-CoV-2 procedures were conducted in a BSL-3 laboratory, adhering to safety protocols approved by the Institut Pasteur Korea’s Biosafety Committee (IPKIBC-RA2020-04).

### 4.6. SARS-CoV-2 VOC Selection and Bioinformatics Analysis

To assess the impact of sequence changes in the Omicron subvariant BA.5 (GenBank accession: ON249995) on T-cell immune epitopes, we performed a comparative analysis with the reference ancestral sequence (NC_045512.2). Sequence alignments are in Supplementary Figures S4 and S5. On 11 July 2022, potential CD4^+^ and CD8^+^ T-cell epitopes were sourced from the Immune Epitope Database (IEDB), following Vita et al. [46]. The IEDB query used these parameters: Epitope: Any, Organism: SARS-CoV-2 (Taxonomy ID: 2697049), Assay Filters: Only positive assays, excluding B cell and major histocompatibility complex (MHC) assays, Host: Homo sapiens, MHC Restriction Type: Class I (CD8) and Class II (CD4).

### 4.7. Xenograft Mice Model

For the tumor xenograft model, NOD/Shi-scid-IL2rγ(null) (NOG) mice, aged 7–9 weeks, were purchased from Jackson Laboratory, Bar Harbor, ME, USA. Mice were housed in a specific pathogen-free (SPF) environment under a 12-h light/dark cycle, 22 °C (±1 °C) temperature, and 55% (±5%) humidity after a one-week quarantine at The Catholic University of Korea’s Laboratory Animal Research Center. HEK293 cells expressing the SARS-CoV-2 nucleocapsid (HEK293-N) (Catalog No. P30920, Innoprot, Spain) or wild-type HEK293 (HEK293-WT) cells were inoculated subcutaneously into the mice at 1-week intervals. The expression levels of HLA-A/B/C and HLA *A02 were confirmed in the HEK293T cells expressing the nucleocapsid. Tumor growth was monitored, and VSTs derived from donors matched for at least one HLA class I allele with the target cells were introduced to evaluate the tumor-suppressive effects of the VSTs. All procedures involving animal experiments were approved by the Institutional Animal Care and Use Committee (IACUC) of The Catholic University of Korea (Approval number: CUMC-2021-0030-01).

### 4.8. Pentamer Assay

SARS-CoV-2-specific MHC class I and II epitopes were detected using 9-mer pentamers for HLA-A02:01, HLA-A03:01, HLA-A11:01, HLA-B04:01, HLA-B07:02, and HLA-B27:05. For MHC class II, 16–18-mer tetramers were used for DRA101:01/DRB101:01, DRA101:01/DRB104:01, and DRA101:01/DRB115:01 (Table 3). LB-DTK-COV19 cells cultured for 21 days were stained with phycoerythrin or allophycocyanin pentamers/tetramers. Surface staining was performed for CD3 (UCHT1), CD56 (TULY56), CD4 (SK3), and CD8 (SK1) markers. Flow cytometric analysis was carried out using a FACSFortessa cytometer (BD Biosciences).

### 4.9. Statistical Analyses

Data are shown as means ± standard deviations. The Mann–Whitney U or Student’s t-test was used for intergroup comparisons, and the Kruskal–Wallis test for multigroup comparisons. Statistical analyses were performed with SPSS version 16.0 (IBM), with *p* < 0.05 considered significant.

## Figures and Tables

**Table 1:** The SARS-CoV-2 Interagency Group (SIG) variant classification scheme defines four classes of SARS-CoV-2 variants *.

WHO Label	Pango Lineage	Date of Designation		
Alpha	B.1.1.7 (RBD mutations: N501Y, A570D) and Q lineages	VOC: 29 December 2020		VBM: 21 September 2021
Beta	B.1.351 (RBD mutations: K417N, E484K, and N501Y) and descendent lineages	VOC: 29 December 2020		VBM: 21 September 2021
Gamma	P.1 and descendent lineages (RBD mutations: K417N/T, E484K, and N501Y)	VOC: 29 December 2020		VBM: 21 September 2021
Delta	B.1.617.2 (RBD mutations: L452R, T478K) and AY lineages	VOC: 15 June 2021		VBM: 14 April 2022
Epsilon	B.1.427 and B.1.429	VOC: 19 March 2021	VOI: 26 February 2021 VOI: 29 June 2021	VBM: 21 September 2021
Eta	B.1.525		VOI: 26 February 2021	VBM: 21 September 2021
Iota	B.1.526		VOI: 26 February 2021	VBM: 21 September 2021
Kappa	B.1.617.1		VOI: 7 May 2021	VBM: 21 September 2021
N/A	B.1.617.3		VOI: 7 May 2021	VBM: 21 September 2021
Zeta	P.2		VOI: 26 February 2021	VBM: 21 September 2021
Mu	B.1.621, B.1.621.1			VBM: 21 September 2021
Omicron	B.1.1.529, BA.1, BA.1.1, BA.2, BA.3, BA.4, BA.5 lineages	VOC: 26 November 2021		
Omicron	BA.2.74, CH.1.1, XBB.1.5, XBB.1.16, XBB.2.3, XBB.1.9.2, XBB.1.9.1, BA.2.86			VBM: 1 September 2023
N/A	Variants containing the F456L spike mutations **		VOI: 1 September 2023	
KP	KP.2, KP.3			VBM: 19 March 2024
JN.1.18	JN.1.18			VBM: 5 April 2024
LB.1	LB.1			VBM: 2 June 2024

* The classification is as per the Centers for Disease Control and Prevention (CDC) dated 30th October 2023. VOC: variants of concern; VOI: variants of interest; VBM: variants being monitored. ** Many lineages have acquired the F456L mutation, and common examples include EG.5, FL.1.5.1, and XBB.1.16.6. SARS-CoV-2: severe acute respiratory syndrome coronavirus 2; WHO: World Health Organization.

**Table 2:** Donor characteristics.

Donor No.	Sex/Age	Vaccine	COVID-19	COVID-19	Days to Recovery	Days from Recovery
				Severity	From Diagnosis	To Cell Production
1	M/29	Unvaccinated	Unexposed	NA	NA	NA
2	F/33	Unvaccinated	Unexposed	NA	NA	NA
3	F/27	Unvaccinated	Unexposed	NA	NA	NA
4	F/23	Unvaccinated	Unexposed	NA	NA	NA
5	M/33	Unvaccinated	Unexposed	NA	NA	NA
6	F/30	Unvaccinated	Unexposed	NA	NA	NA
7	M/32	Unvaccinated	Unexposed	NA	NA	NA
8	F/27	Unvaccinated	Unexposed	NA	NA	NA
9	F/27	Unvaccinated	Unexposed	NA	NA	NA
10	M/25	Unvaccinated	Unexposed	NA	NA	NA
11	F/29	Unvaccinated	Unexposed	NA	NA	NA
12	F/64	Unvaccinated	Unexposed	NA	NA	NA
13	F/39	Unvaccinated	Unexposed	NA	NA	NA
14	M/26	Unvaccinated	Unexposed	NA	NA	NA
15	M/25	Unvaccinated	Unexposed	NA	NA	NA
16	M/65	Unvaccinated	Unexposed	NA	NA	NA
17	F/36	Vaccinated	Unexposed	NA	NA	NA
18	M/46	Vaccinated	Unexposed	NA	NA	NA
19	F/36	Vaccinated	Unexposed	NA	NA	NA
20	M/28	Vaccinated	Unexposed	NA	NA	NA
21	F/24	Vaccinated	Unexposed	NA	NA	NA
22	M/51	Vaccinated	Unexposed	NA	NA	NA
23	M/27	Vaccinated	Unexposed	NA	NA	NA
24	F/29	Vaccinated	Unexposed	NA	NA	NA
25	F/29	Vaccinated	Unexposed	NA	NA	NA
26	F/61	Unvaccinated	Recovered	Mild	23	103
27	M/61	Unvaccinated	Recovered	Severe	31	90
28	M/43	Unvaccinated	Recovered	Mild	14	113
29	F/58	Unvaccinated	Recovered	Mild	26	87
30	F/60	Unvaccinated	Recovered	Mild	15	78
31	M/23	Unvaccinated	Recovered	Mild	Unknown	Unknown
32	M/23	Unvaccinated	Recovered	Mild	Unknown	Unknown
33	F/50	Unvaccinated	Recovered	Mild	14	Unknown
34	M/33	Vaccinated	Recovered	Mild	14	Unknown
35	M/31	Vaccinated	Recovered	Mild	14	7
36	F/25	Vaccinated	Recovered	Mild	14	7
37	F/69	Vaccinated	Not within 6 mo	Moderate	NA	NA
38	F/21	Vaccinated	Not within 6 mo	Moderate	NA	NA
39	M/46	Vaccinated	Not within 6 mo	Moderate	NA	NA
40	F/81	Vaccinated	Not within 6 mo	Moderate	NA	NA

COVID 19: coronavirus disease; F: female; M: male; NA: not applicable.

**Table 3:** SARS-CoV-2 pentamer and tetramer sequences.

SARS-CoV-2 Protein	Pentamer Tetramer	aa Start	aa Stop	MHC Class Allele
Spike	YLQPRTFLL	269	277	HLA-A*02:01
Spike	KCYGVSPTK	378	386	HLA-A*03:01
Spike	ALNTLVKQL	940	948	HLA-A*02:01
Spike	VLNDILSRL	958	966	HLA-A*02:01
Spike	LITGRLQSL	996	1004	HLA-A*02:01
Spike	RLQSLQTYV	1000	1008	HLA-A*02:01
Spike	NLNESLIDL	1174	1182	HLA-A*02:01
Spike	QYIKWPWYI	1205	1213	HLA-A*24:02
Spike	FIAGLIAIV	1220	1228	HLA-A*02:01
Spike	TRFQTRFQTLLALHRSYLT	236	254	DRA1*01:01/DRB1*01:01
Spike	GAALQIPFAMQMAYRF	873	888	DRA1*01:01/DRB1*01:01
Spike	MAYRFNGIGVTQNVLY	884	899	DRA1*01:01/DRB1*01:01
Spike	QALNTLVKQLSSNFGAI	939	955	DRA1 *01:01/DRB1 *04:01
Spike	QLIRAAEIRASANLAATK	993	1010	DRA1*01:01/DRB1*01:01
Nucleocapsid	QRNAPRITF	9	17	HLA-B*27:05
Nucleocapsid	SPRWYFYYL	105–113	113	HLA-B*07:02
Nucleocapsid	LLLDRLNQL	223	231	HLA-A*02:01
Nucleocapsid	MEVTPSGTWL	322	330	HLA-B*04:01
Nucleocapsid	KTFPPTEPK	362	370	HLA-A*03:01

MHC: major histocompatibility complex; SARS-CoV-2: severe acute respiratory syndrome coronavirus 2.

## Data Availability

The amino acid sequences used in this study were obtained from the National Center for Biotechnology Information (NCBI) database. Accession numbers or specific identifiers for these sequences are available in Section 4.

## Supplementary Materials

The following supporting information can be downloaded at: https://www.mdpi.com/article/10.3390/ijms251910512/s1.
