# Supplementary material for: SARS-CoV-2-Specific T-Cell as a Potent Therapeutic Strategy against Immune Evasion of Emerging COVID-19 Variants
Source: Int J Mol Sci. 2024 Sep 29;25(19):10512. doi: 10.3390/ijms251910512 (PMC11477143; doi:10.3390/ijms251910512)
Supplement: Supplementary file 1 [file ijms-25-10512-s001.zip › ijms-3218655-supplementary.pdf]

Supplementary Material

SARS-CoV-2-Specific T-Cell as a Potent Therapeutic Strategy against Immune Evasion of Emerging COVID-19 Variants

S1.1. Supplementary Figures

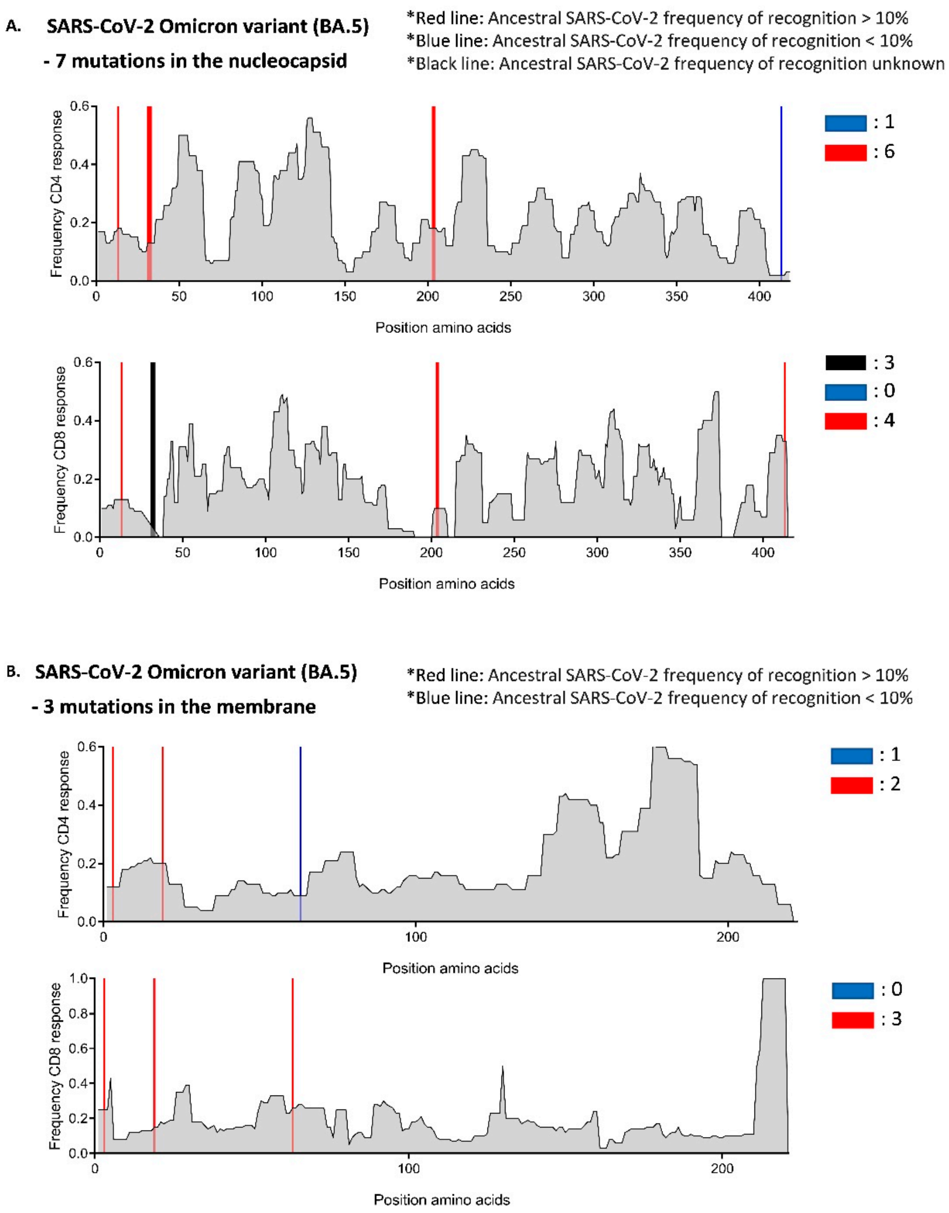

Supplementary Figure S1. VSTs educated by the ancestral SARS-CoV-2 strain recognize conserved nucleocapsid and membrane epitopes in the Omicron variant. The expected response frequency for the nucleocapsid and membrane-mutation domain of Omicron BA.5. (A) Analysis of nucleocapsid-protein epitopes in the Omicron sub-variant (BA.5) targeted by $CD4^+$ T cells (conservation rate: 82%) and recognized by $CD8^+$ T cells (conservation rate: 85%). (B) Analysis of membrane-protein epitopes in the Omicron sub-variant (BA.5) targeted by $CD4^+$ T cells (conservation rate: 96%) and recognized by $CD8^+$ T cells (conservation rate: 96%). SARS-CoV-2: severe acute respiratory syndrome coronavirus-2; VST: virus-specific T cell.

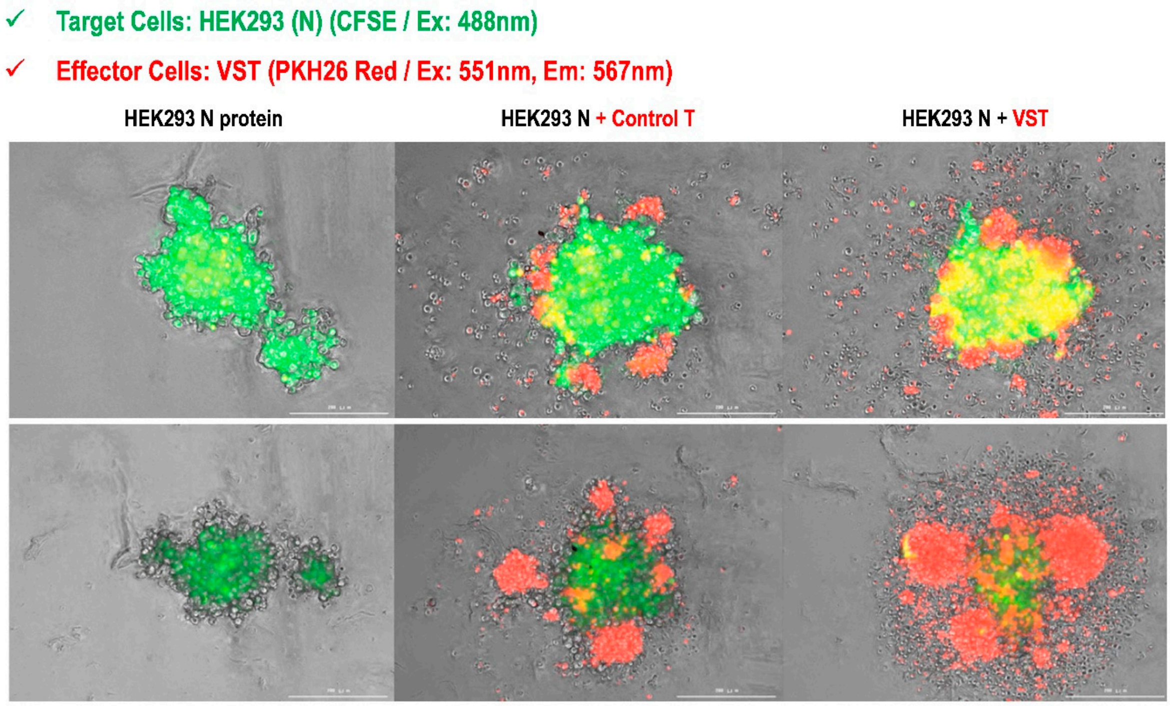

Supplementary Figure S2. Evaluation of VST cytotoxicity against SARS-CoV-2 N protein-expressing cells. Live cell imaging of VSTs on HEK293 N cells expressing the SARS-CoV-2 N protein. CFSE-labelled HEK293 N cell spheroids were co-cultured with PKH26 Red-labeled control T cells (Control T) and PKH26 Red-labeled SARS-CoV-2-specific T cells (VSTs) for 24 h. CFSE: carboxyfluorescein succinimidyl ester; N: nucleocapsid; SARS-CoV-2: severe acute respiratory syndrome coronavirus 2; VST: virus-specific T cell.

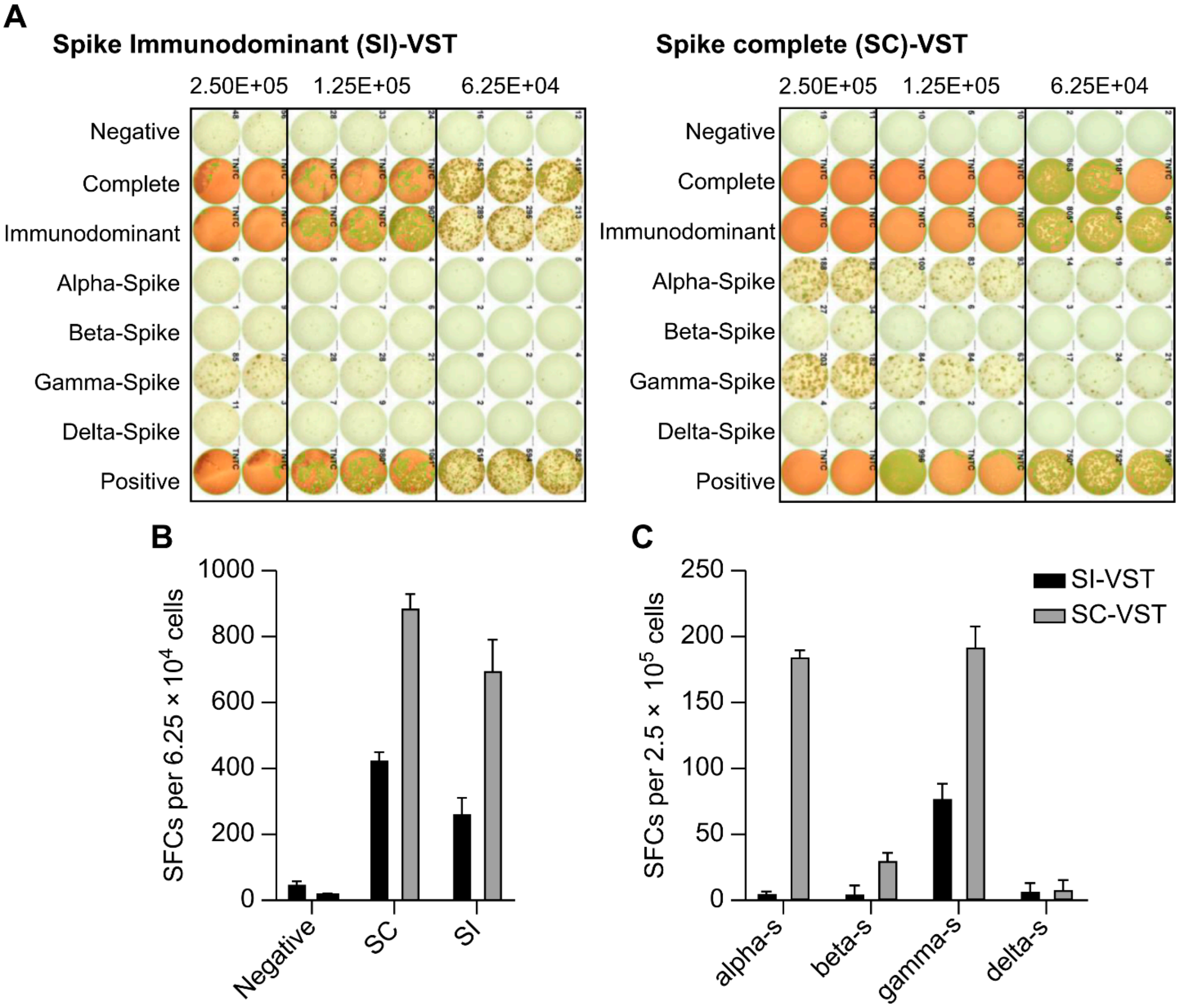

Supplementary Figure S3. Comparison of multiple epitope recognition abilities between VSTs stimulated by spike immunodominant peptide and VSTs stimulated by spike complete peptide. Based on ELISPOT analysis, the multiple epitope recognition abilities were compared between VSTs stimulated by spike immunodominant (SI) peptide and VSTs stimulated by spike complete (SC) peptide. (A) VST response against SARS-CoV-2 antigens. Data in box plots show spot-forming cells (SFCs). (B) Summarized results from 6.25×10^4 cells and (C) 2.5×10^5 cells. SARS-CoV-2: severe acute respiratory syndrome coronavirus-2; VST: virus-specific T cell.

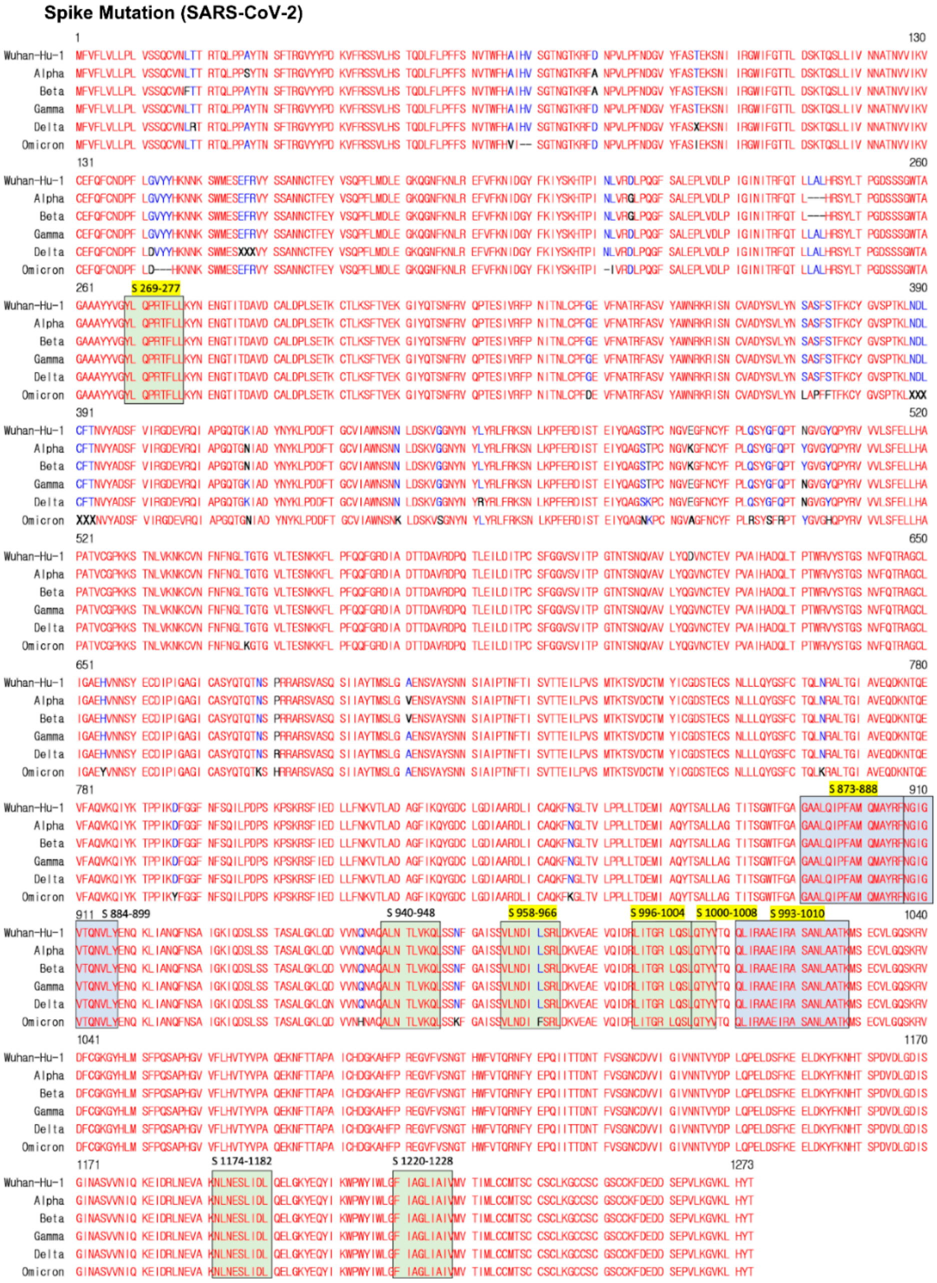

Supplementary Figure S4. SARS-CoV-2 spike mutation bioinformatic analysis. Changes in the subvariant (Alpha, Beta, Gamma, Delta, Omicron) spike sequences are shown compared to the reference ancestral SARS-CoV-2-1 spike sequence. The MHC class I and MHC class II pentamer sequences used in the pentamer assay are indicated with green and purple boxes, respectively. MHC: major histocompatibility complex; SARS-CoV-2: severe acute respiratory syndrome coronavirus 2.

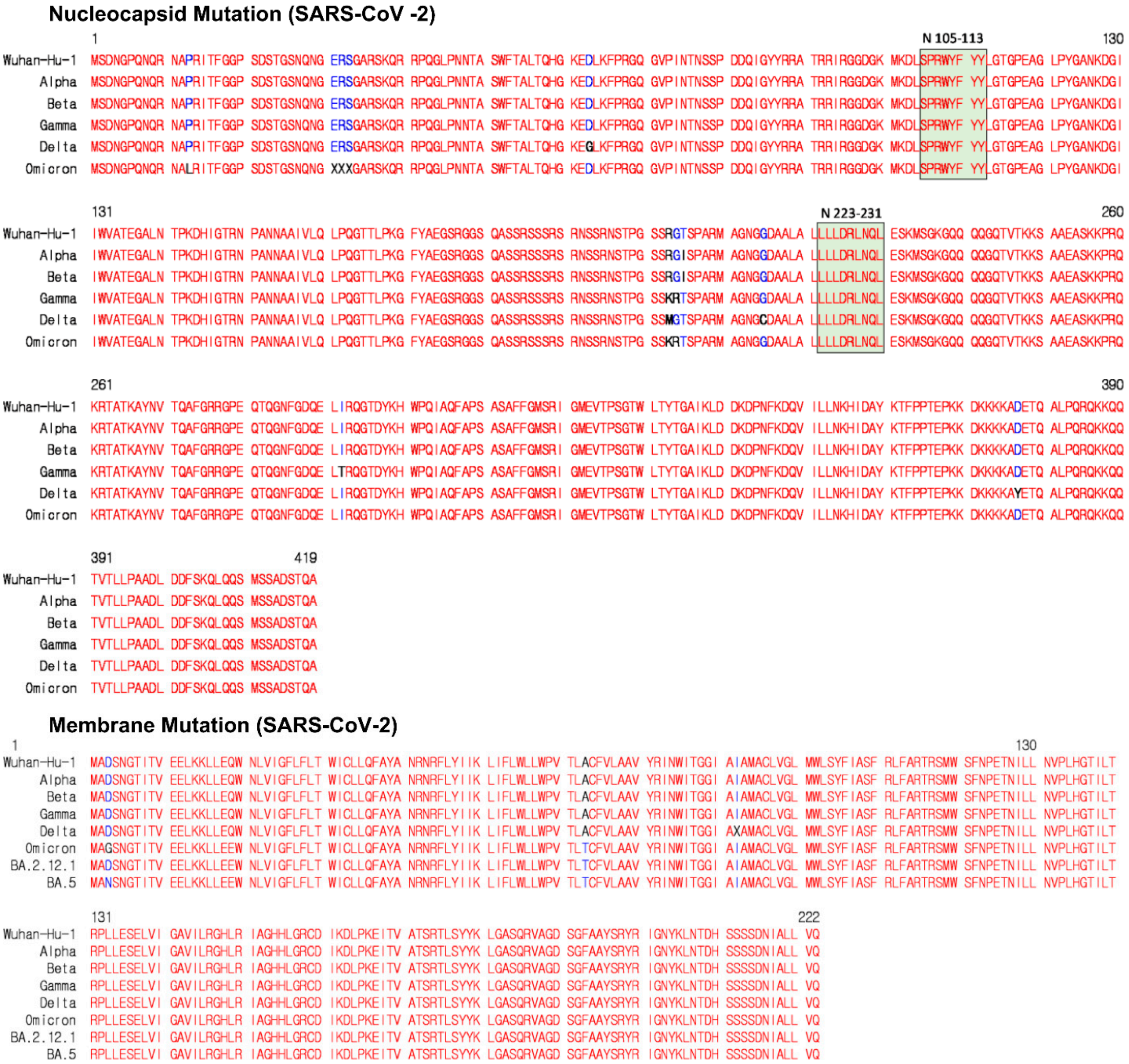

Supplementary Figure S5. SARS-CoV-2 nucleocapsid and membrane mutation bioinformatic analysis. Changes in the subvariant (Alpha, Beta, Gamma, Delta, Omicron) nucleocapsid and membrane sequences are shown compared to the reference ancestral SARS-CoV-2-1 nucleocapsid and membrane sequence. The MHC class I pentamer sequence used in the pentamer assay is indicated by a green box. MHC: major histocompatibility complex; SARS-CoV-2: severe acute respiratory syndrome coronavirus 2.

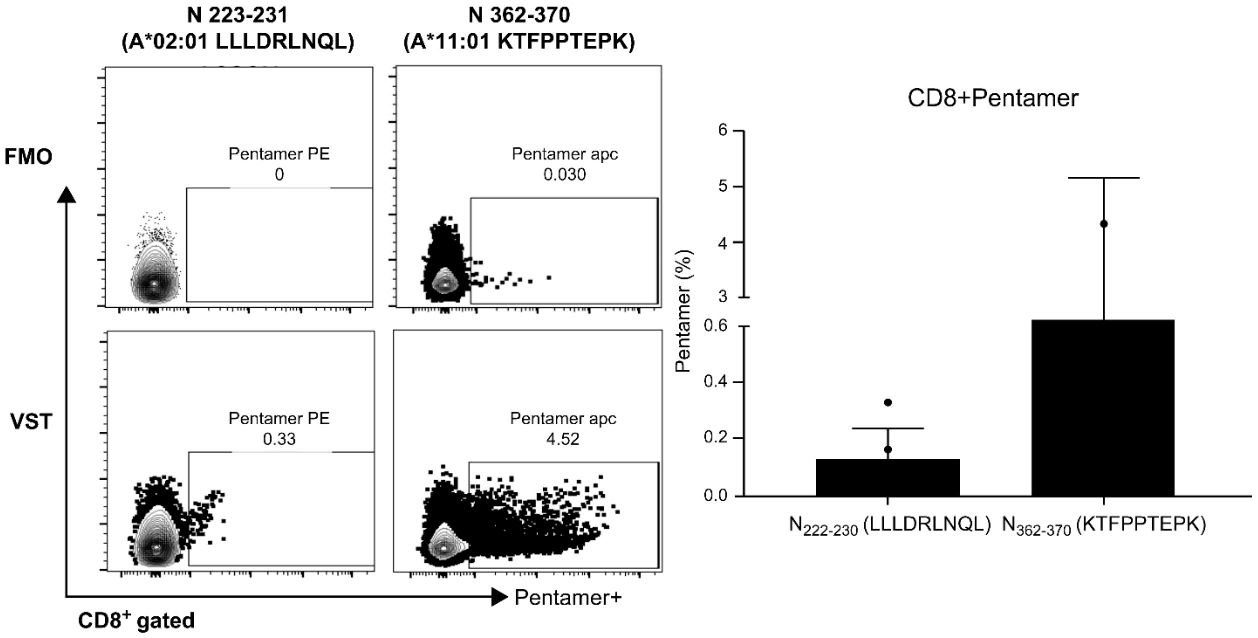

Supplementary Figure S6. VSTs educated by the ancestral SARS-CoV-2 strain recognize conserved nucleocapsid epitopes in the Omicron variant. Representative data showcasing the pronounced reactivity of VSTs against two specific Omicron BA.5 nucleocapsid-protein immunogenic epitopes, determined by Pentamer analysis. A bar graph consolidates the results from 10 donors. APC: allophycocyanin; FMO: fluorescence minus one; N: nucleocapsid; PE: phycoerythrin; SARS-CoV-2: severe acute respiratory syndrome coronavirus 2; VST: virus-specific T cell.

S1.2. Supplementary Table

Supplementary Table S1. HLA types of donors

Donor	HLA-A		HLA-B		HLA-C		HLA-DRB	
1	*02:01	*33:03	*15:07	*58:01	*03:02	*03:03	*04:03	*13:02
2	*24:02	*30:01	*07:02	*40:06	*07:02	*08:01	*01:01	*08:03
3	*24:02	*30:01	*13:02	*40:02	*03:04	*06:02	*07:01	*11:01
4	*24:02	*26:02	*51:01	*54:01	*07:02	*14:02	*11:01	*14:05
5	*02:01	*02:01	*07:02	*15:11	*03:03	*07:02	*09:01	*15:01
6	*24:02	*33:03	*40:06	*44:03	*01:02	*14:03	*09:01	*13:02
7	*02:06	*33:03	*44:03	*54:01	*01:02	*14:03	*13:02	*15:01
8	*02:07	*24:02	*15:11	*46:01	*01:02	*03:03	*09:01	*09:01
9	*02:01	*24:02	*27:05	*40:06	*01:02	*02:02	*14:05	*15:02
10	*24:02	*24:02	*15:07	*56:01	*01:02	*03:03	*04:03	*14:54
11	*02:06	*30:01	*13:02	*51:01	*06:02	*14:02	*04:05	*11:01
12	*24:02	*24:01	*51:01	*54:01	*01:02	*14:02	*04:05	*12:01
13	*24:02	*33:03	*44:03	*51:01	*07:06	*14:02	*07:01	*12:01
14	*02:06	*24:02	*07:02	*54:01	*01:02	*07:02	*01:01	*04:05
15	*02:01	*11:01	*40:02	*56:01	*01:02	*03:04	*04:05	*09:01
16	*02:01	*24:02	*13:01	*51:01	*03:04	*14:02	*09:01	*14:54
17	*24:02	*26:01	*51:01	*54:01	*07:02	*14:02	*11:01	*14:05
18	*02:01	*02:06	*37:01	*38:02	*06:02	*07:02	*08:03	*10:01
19	*02:01	*31:01	*07:02	*51:02	*07:02	*15:02	*01:01	*08:02
20	*02:01	*31:01	*15:01	*51:01	*04:01	*14:02	*04:04	*04:06
21	*02:01	*24:02	*15:18	*40:06	*07:04	*08:01	*04:05	*04:10
22	*24:02	*33:03	*51:01	*58:01	*03:02	*14:02	*13:02	*14:05
23	*11:01	*24:02	*15:01	*51:01	*04:01	*14:02	*04:06	*14:05
24	*02:01	*02:07	*15:01	*15:01	*04:01	*04:01	*04:06	*14:54
25	*01:01	*24:02	*35:01	*35:01	*03:03	*04:01	*04:07	*11:01
26	*02:01	*33:03	*55:02	*58:01	*03:02	*03:03	*12:01	*13:02
27	*24:02	*26:02	*15:07	*51:02	*03:03	*15:02	*04:03	*09:01
28	*11:01	*24:02	*54:01	*54:01	*01:02	*01:02	*04:05	*08:03
29	*02:01	*11:01	*51:01	*15:11	*03:03	*15:02	*04:05	*14:05
30	*02:01	*30:01	*40:06	*44:03	*03:03	*07:06	*07:01	*14:03
31	*24:02	*33:03	*35:01	*44:03	*03:04	*07:06	*04:03	*07:01
32	*02:01	*03:01	*13:01	*27:05	*02:02	*03:04	*04:05	*15:02
33	*24:02	*33:03	*40:06	*58:01	*03:02	*08:01	*03:01	*04:10
34	*02:01	*33:03	*44:03	*54:01	*01:02	*14:03	*08:03	*13:02
35	*02:01	*24:04	*15:01	*40:03	*01:02	*03:01	*09:01	*15:01
36	*02:01	*33:03	*15:18	*58:01	*03:02	*07:04	*04:03	*13:02
37	*26:02	*31:01	*15:18	*40:06	*07:04	*08:01	*04:01	*09:01
38	*24:02	*26:03	*15:01	*35:01	*03:03	*03:03	*09:01	*15:01
39	*11:01	*31:01	*15:18	*40:02	*03:04	*08:01	*04:51	*08:02
40	*24:02	*30:01	*52:01	*55:04	*03:03	*12:02	*04:05	*15:02

HLA: human leukocyte antigen; RBD: receptor-binding domain.
